# Tracking the amphibian pathogens *Batrachochytrium dendrobatidis* and *Batrachochytrium salamandrivorans* using a highly specific monoclonal antibody and lateral‐flow technology

**DOI:** 10.1111/1751-7915.12464

**Published:** 2016-12-19

**Authors:** Michael J. Dillon, Andrew E. Bowkett, Michael J. Bungard, Katie M. Beckman, Michelle F. O'Brien, Kieran Bates, Matthew C. Fisher, Jamie R. Stevens, Christopher R. Thornton

**Affiliations:** ^1^ BiosciencesUniversity of ExeterGeoffrey Pope BuildingExeterEX4 4QDUK; ^2^ Whitley Wildlife Conservation TrustPaigntonTQ4 7EUUK; ^3^ Wildfowl & Wetlands TrustSlimbridgeGL2 7BTUK; ^4^ Department of Infectious Disease EpidemiologyImperial College LondonLondonSW7 2AZUK

## Abstract

The fungus *Batrachochytrium dendrobatidis* (*Bd*) causes chytridiomycosis, a lethal epizootic disease of amphibians. Rapid identification of the pathogen and biosecurity is essential to prevent its spread, but current laboratory‐based tests are time‐consuming and require specialist equipment. Here, we describe the generation of an IgM monoclonal antibody (mAb), 5C4, specific to *Bd* as well as the related salamander and newt pathogen *Batrachochytrium salamandrivorans* (*Bsal*). The mAb, which binds to a glycoprotein antigen present on the surface of zoospores, sporangia and zoosporangia, was used to develop a lateral‐flow assay (LFA) for rapid (15 min) detection of the pathogens. The LFA detects known lineages of *Bd* and also *Bsal*, as well as the closely related fungus *Homolaphlyctis polyrhiza*, but does not detect a wide range of related and unrelated fungi and oomycetes likely to be present in amphibian habitats. When combined with a simple swabbing procedure, the LFA was 100% accurate in detecting the water‐soluble 5C4 antigen present in skin, foot and pelvic samples from frogs, newts and salamanders naturally infected with *Bd* or *Bsal*. Our results demonstrate the potential of the portable LFA as a rapid qualitative assay for tracking these amphibian pathogens and as an adjunct test to nucleic acid‐based detection methods.

## Introduction

Amphibians have inhabited the planet for over 350 million years and have withstood four of the past mass extinction events (Wake and Vredenburg, 2008). Since 1980, however, more than one‐third of the world's amphibians have been experiencing rapid population declines (Stuart *et al*., 2004), with more than 2000 species classified as extremely vulnerable or critically endangered (IUCN, 2016). While it can be argued that much vertebrate life on Earth is experiencing losses of biodiversity, amphibians are declining disproportionally faster than both mammals and birds combined (Stuart *et al*., 2004). This is a serious concern as amphibians play myriad roles in ecosystem services, contributing to aquatic bioturbation, nutrient cycling and controlling pests (Hocking and Babbitt, 2014).

There are a number of factors contributing to global amphibian decline, including habitat reduction, overexploitation and infectious diseases (Hocking and Babbitt, 2014). Amphibian population sizes are shrinking due to urbanization (Cushman, 2006) and, of the surviving species, many are hunted for human consumption or as part of the international pet trade (Garner *et al*., 2009; Herrel and van der Meijden, 2014). Importantly, novel emerging pathogens such as ranavirus and the fungal species *Batrachochytrium dendrobatidis* (*Bd*) and *B. salamandrivorans* (*Bsal*) (Granoff *et al*., 1966; Berger *et al*., 1998; Stuart *et al*., 2004; Skerratt *et al*., 2007; Martel *et al*., 2013) are now known to be important proximal drivers of global losses to amphibian biodiversity.

*Batrachochytrium dendrobatidis*, a member of the primitive fungal phylum Chytridiomycota (James *et al*., 2006), was the first recognized aetiological agent of chytridiomycosis, a lethal skin disease of amphibians (Berger *et al*., 1998). The pathogen was not discovered until 1999, almost 20 years after amphibian decline was first noted (Berger *et al*., 1998), and since then is thought to have contributed to the extinction of over 200 amphibian species and the population declines of many more (Skerratt *et al*., 2007). As an aquatic organism, it has two life stages, a substrate‐independent phase characterized by motile zoospores and a substrate‐dependent phase characterized by encysted sporangia (Berger *et al*., 2005). Motile zoospores occur in freshwater ponds and streams where they are attracted to keratinized tissues found in frogs and tadpoles, where they encyst and infect the amphibian host (Moss *et al*., 2008). *Bsal* is a more recently discovered chytrid that is the sister species to *Bd* and also causes chytridiomycosis in amphibians, specifically in salamanders and newts (Martel *et al*., 2013).

Infection of the host by *Bd* induces hyperplasia and hyperkeratosis, causing osmotic imbalances and eventually cardiac arrest (Voyles *et al*., 2009). Symptoms of infection are ambiguous and include loss of righting reflex, suppression of appetite and lethargy (Berger *et al*., 1999a; Voyles *et al*., 2009). As such, it is extremely difficult to diagnose infection without the use of invasive biopsy and histology and/or quantitative polymerase chain reaction (qPCR) of skin swabs (Berger *et al*., 1999b; Hyatt *et al*., 2007). These techniques are time‐consuming, require skilled personnel and are restricted to laboratories equipped with sophisticated and expensive equipment, so are ill suited to the rapid identification of the pathogen in resource‐limited settings.

Hybridoma technology allows the generation of highly specific monoclonal antibodies that are able to differentiate between different genera and species of fungi or even spores and hyphae of the same species (Thornton, 2008, 2009; Davies and Thornton, 2014; Thornton *et al*., 2015; Al‐Maqtoofi and Thornton, 2016). Monoclonal antibodies have been used in a number of rapid point‐of‐care lateral‐flow assays (LFA) to successfully detect the presence of fungal or oomycete pathogens of vertebrates *in vivo* including *Cryptococcus neoformans* (Kozel and Bauman, 2012), *Candida albicans* (Marot‐Leblond *et al*., 2004), *Pythium insidiosum* (Krajaejun *et al*., 2009) and *Aspergillus* spp. (Thornton, 2008).

The purpose of this study is to report the development of a murine mAb (clone 5C4) specific to *Bd*,* Bsal* and the non‐pathogenic chytrid *Homolaphlyctis polyrhiza*, which has recently been grouped in molecular phylogenies as a sister taxa to *Bd* (Longcore *et al*., 2011). Using the mAb, we have developed a LFA for rapid (15 min) detection of these fungi. The LFA, which recognizes a diagnostic water‐soluble glycoprotein antigen detectable in skin swabs of animals infected with *Bd* and *Bsal*, is a simple, portable, diagnostic test that holds enormous potential for tracking these pathogens in their natural environments.

## Results

### Production of hybridoma cell lines, isotyping of mAb and specificity

Four BALB/c mice were immunized with *Bd*‐global panzootic lineage (*Bd*‐GPL) JEL423, a member of the hypervirulent *Bd*‐GPL. Three mice were selected for hybridoma generation based on serum antibody titres. The resultant 5760 hybridoma cell lines were screened by ELISA for recognition of the immunogen, and a single mAb, designated 5C4, was selected for further studies based on its strength of immunoreactivity. The mAb belongs to the immunoglobulin class M (IgM).

**Figure 1:**
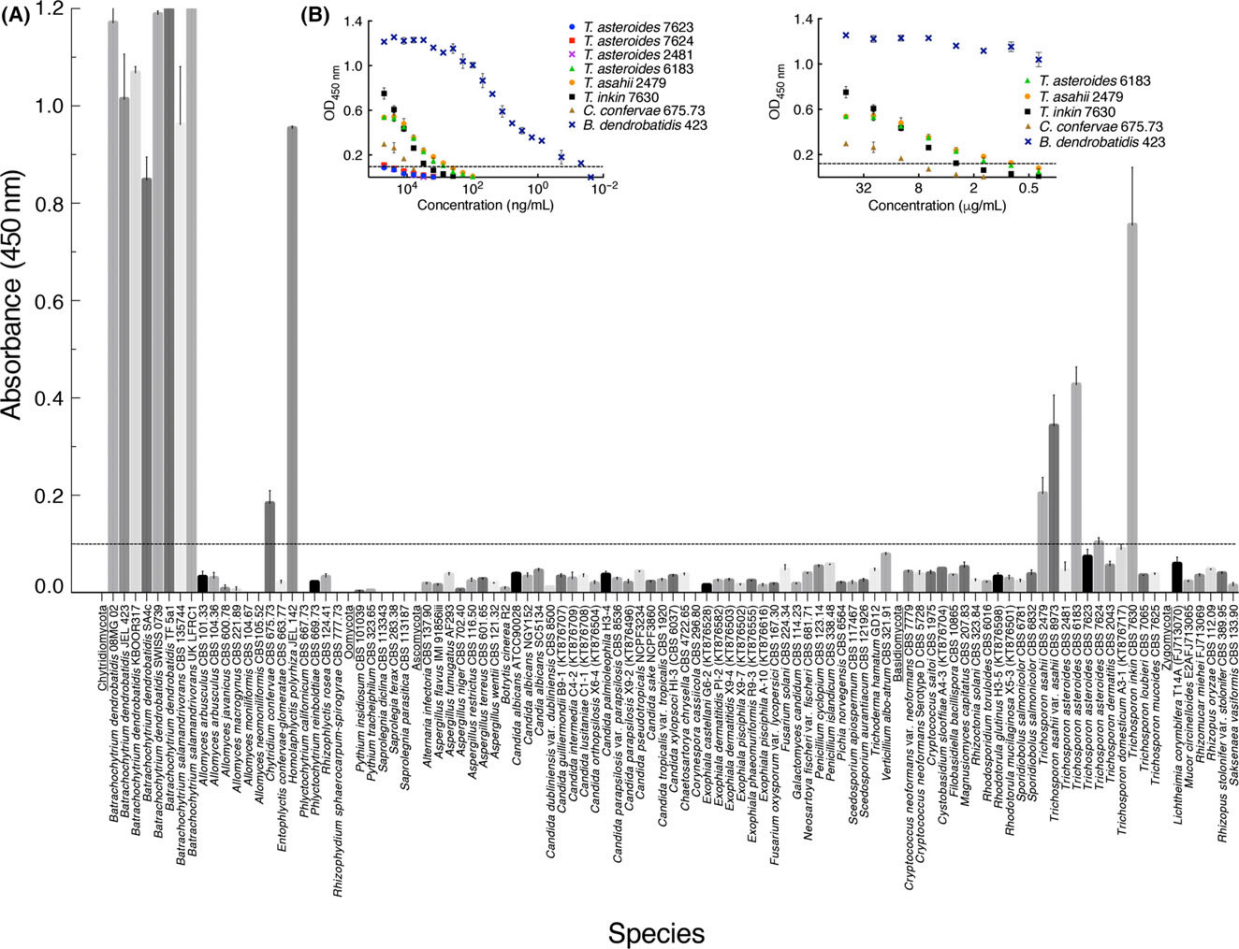
Specificity of 5C4 determined by ELISA tests of surface washings containing water‐soluble antigens from *Batrachochytrium* species and related and unrelated fungi and oomycetes. (A) ELISA absorbance values at 450 nm for antigens from *Batrachochytrium dendrobatidis*,* Batrachochytrium salamandrivorans*, the sister chytrid *Homolaphlyctis polyrhiza* and other related and unrelated yeasts, yeast‐like fungi, moulds and oomycetes in the Chytridiomycota, Oomycota, Ascomycota, Basidiomycota and Zygomycota. Bars are the means of three biological replicates ± standard errors, and the threshold absorbance value for detection of antigen in ELISA is ≥ 0.100 (indicated by line on graph). Wells were coated with 250 μg protein mL^−1^ buffer. Cross‐reactivity of 5C4 with the related chytrid fungus *Chytridium confervae*, and non‐related species of the yeast‐like fungus *Trichosporon*, is evident at this concentration of protein. (B) ELISA for antigens from *B. dendrobatidis* isolate *Bd*‐JEL423, the related cross‐reactive chytrid *C. confervae* and unrelated cross‐reactive species of *Trichosporon*. While cross‐reaction of 5C4 with *C. confervae* and *Trichosporon* is shown at 250 μg protein mL^−1^, it is eliminated at 200 ng mL^−1^ (compared with loss of reactivity with *Bd*‐JEL423 antigen at 0.05 ng mL^−1^).

MAb 5C4 was tested in ELISA for specificity against a panel of geographically distinct *Bd* lineages and against two isolates of the related amphibian pathogen *Bsal*, to ensure that it recognizes genotypically distinct lineages of the two fungi. Furthermore, 5C4 was tested against an extensive collection of related and unrelated fungi and fungal‐like organisms belonging to the chytridiomycota, zygomycota, ascomycota, basidiomycota and oomycota, present in both aquatic and terrestrial environments (Table S1). MAb 5C4 reacted with all lineages of *Bd* that we tested (*Bd*‐GPL, *Bd*‐CAPE, *Bd*‐SWISS and *Bd*‐ASIA), with the two isolates of *Bsal*, and also with the sister chytrid *H. polyrhiza* JEL142 (Fig. 1A). The mAb did not react with other members of the *Rhizophydiales* (*Entophlyctis*,* Rhizophydium*,* Rhizophlyctis*), with the chytrids *Allomyces* and *Phlyctochytrium*, with the oomycetes *Pythium* and *Saprolegnia*, nor with a wide range of yeasts, yeast‐like fungi and filamentous moulds across the four phyla of fungi tested (Fig. 1A). The mAb did however cross‐react with the chytrid *Chytridium confervae* and with yeast‐like *Trichosporon* spp. (Fig. 1A). However, while cross‐reactivity of 5C4 with *C. confervae* and *Trichosporon* species was demonstrated at 250 μg protein mL^−1^, reactivity with these fungi was eliminated at 200 ng protein mL^−1^ (compared with loss of reactivity with *Bd*‐GPL JEL423 antigens at 0.05 ng mL^−1^; Fig. 1B).

### Western blotting of the 5C4 antigen and epitope characterization

**Figure 2:**
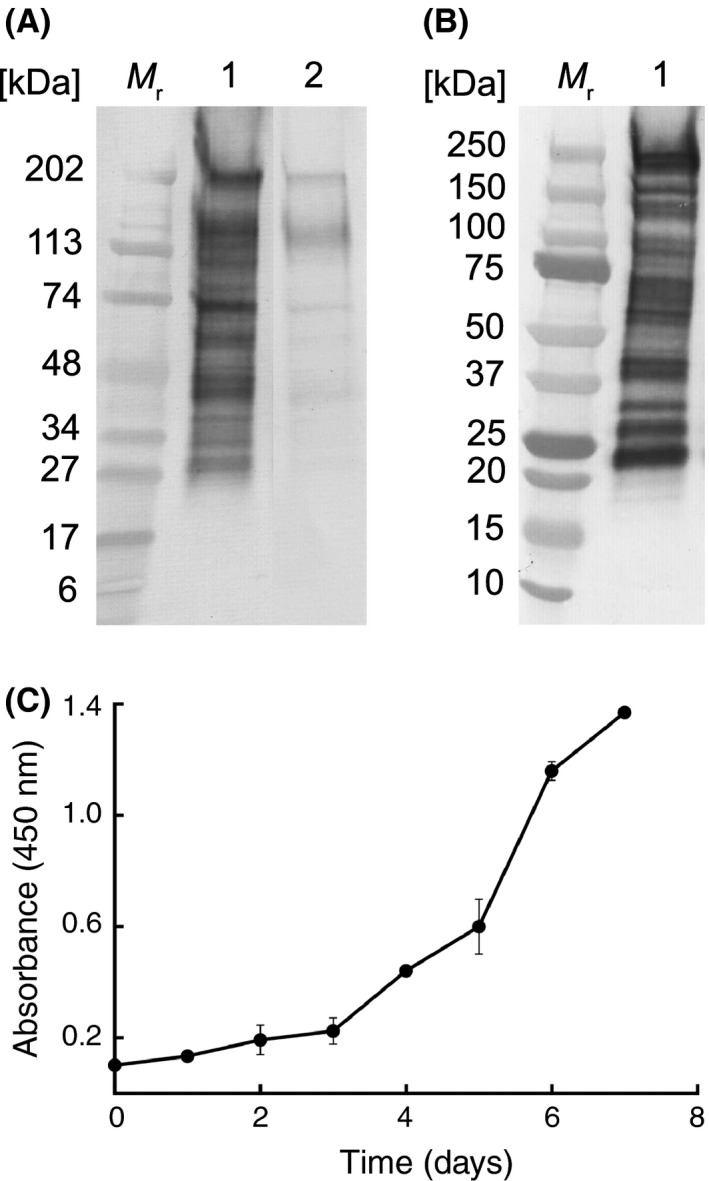
Characterization of the 5C4 antigen and extracellular antigen production. (A) Western immunoblot with 5C4 using the *Bd*‐GPL JEL423 immunogen and following treatment of PVDF membranes with acetate buffer only (lane 1) or with periodate (lane 2). The reduction of immunoreactivity of 5C4 with glycoproteins between ∼27 and ∼220 kDa following periodate treatment shows that the antibody binds to carbohydrate moieties containing vicinal hydroxyl groups. Wells were loaded with 1.6 μg protein. *M*_r_ denotes molecular weight in kDa. (B) Western immunoblot with 5C4 using washed, lyophilized zoospores of *Bd*‐GPL JEL423. Wells were loaded with 1.6 μg protein. *M*_r_ denotes molecular weight in kDa. (C) ELISA absorbance values at 450 nm for extracellular 5C4‐reactive antigens present in liquid cultures of *Bd*‐GPL JEL423. Each point is the mean of three biological ± standard errors. The increase in absorbance values over the 7‐day sampling period shows that the antigen is shed into the external environment during growth and differentiation of the fungus.

Gel electrophoresis and Western blotting of *Bd*‐GPL JEL423 antigens showed that 5C4 recognizes a glycoprotein antigen with a molecular weight of between ~27 and ~220 kDa present in the immunogen and in washed zoospore preparations (Fig. 2A and B), while ELISA tests showed it is released extracellularly during fungal development (Fig. 2C). *Bd*‐GPL JEL423 antigens were subjected to chemical (Table 1 and Fig. 2A), heat (Table 2) and enzymatic (Table 3) treatments in order to characterize the epitope bound by 5C4. Reductions in mAb binding following chemical digestion of an antigen with periodate show that its epitope is carbohydrate and contains vicinal hydroxyl groups. The reduction in 5C4 binding in ELISA following 16 h of periodate treatment of immobilized antigens (Table 1) indicates that its epitope contains carbohydrate moieties. This was further tested using periodate treatment of antigens in Western blots. The reduced binding of 5C4 to its antigen in periodate‐treated blots compared with acetate‐treated controls (Fig. 2A) confirmed its recognition of a carbohydrate epitope. Reductions in mAb binding following heat treatment show that the epitope is heat labile. There was no significant reduction in 5C4 binding over 60 min of heating, showing that its epitope is heat stable (Table 2). Reductions in mAb binding following treatment with pronase shows that its epitope consists of protein, while reductions with trypsin indicate a protein epitope containing positively charged lysine and arginine side‐chains. The lack of reduction in 5C4 binding following digestion of immobilized antigen with trypsin and pronase (Table 3) shows that it does not bind to a protein epitope. Taken together, these results indicate that 5C4 binds to an extracellular antigen and that its epitope is a heat‐stable carbohydrate containing vicinal hydroxyl groups.

**Table 1:** Absorbance values from ELISA tests with mAb 5C4 using periodate‐treated *Bd*‐GPL JEL423 antigens

Time (h)	Absorbance (450 nm)^a^	
	Periodate	Control
0	1.273 ± 0.075	1.313 ± 0.044
1	1.324 ± 0.036	1.388 ± 0.025
2	1.314 ± 0.021	1.365 ± 0.048
3	1.283 ± 0.048	1.326 ± 0.033
4	1.286 ± 0.006	1.357 ± 0.011
16	0.291 ± 0.008^b^	1.313 ± 0.044

a Each value is the mean of three replicate samples.

b Absorbance value significantly different (*P* < 0.001) to matched control using ANOVA.

**Table 2:** Absorbance values from ELISA tests with mAb 5C4 using heat‐treated *Bd*‐GPL JEL423 antigens

Time (min)	Absorbance (450 nm)^a^	
	Heat	Control
0	1.145 ± 0.111	1.233 ± 0.031
10	1.317 ± 0.057	1.288 ± 0.038
20	1.306 ± 0.047	1.305 ± 0.043
30	1.289 ± 0.095	1.314 ± 0.067
40	1.298 ± 0.063	1.356 ± 0.039
50	1.327 ± 0.068	1.325 ± 0.085
60	1.229 ± 0.025	1.234 ± 0.045

a Each value is the mean of three replicate samples.

**Table 3:** Absorbance values from ELISA tests with mAb 5C4 using trypsin‐ or pronase‐treated *Bd*‐GPL JEL423 antigens

Temp (°C)	Absorbance (450 nm)^a^			
	Trypsin	Control	Pronase	Control
4	1.066 ± 0.128	1.120 ± 0.044	1.177 ± 0.040	1.184 ± 0.048
37	1.150 ± 0.076	1.173 ± 0.070	1.112 ± 0.052	1.230 ± 0.036

a Each value is the mean of three replicate samples.

### Spatio‐temporal localization of antigen by ELISA, immunofluorescence and immunogold electron microscopy

**Figure 3:**
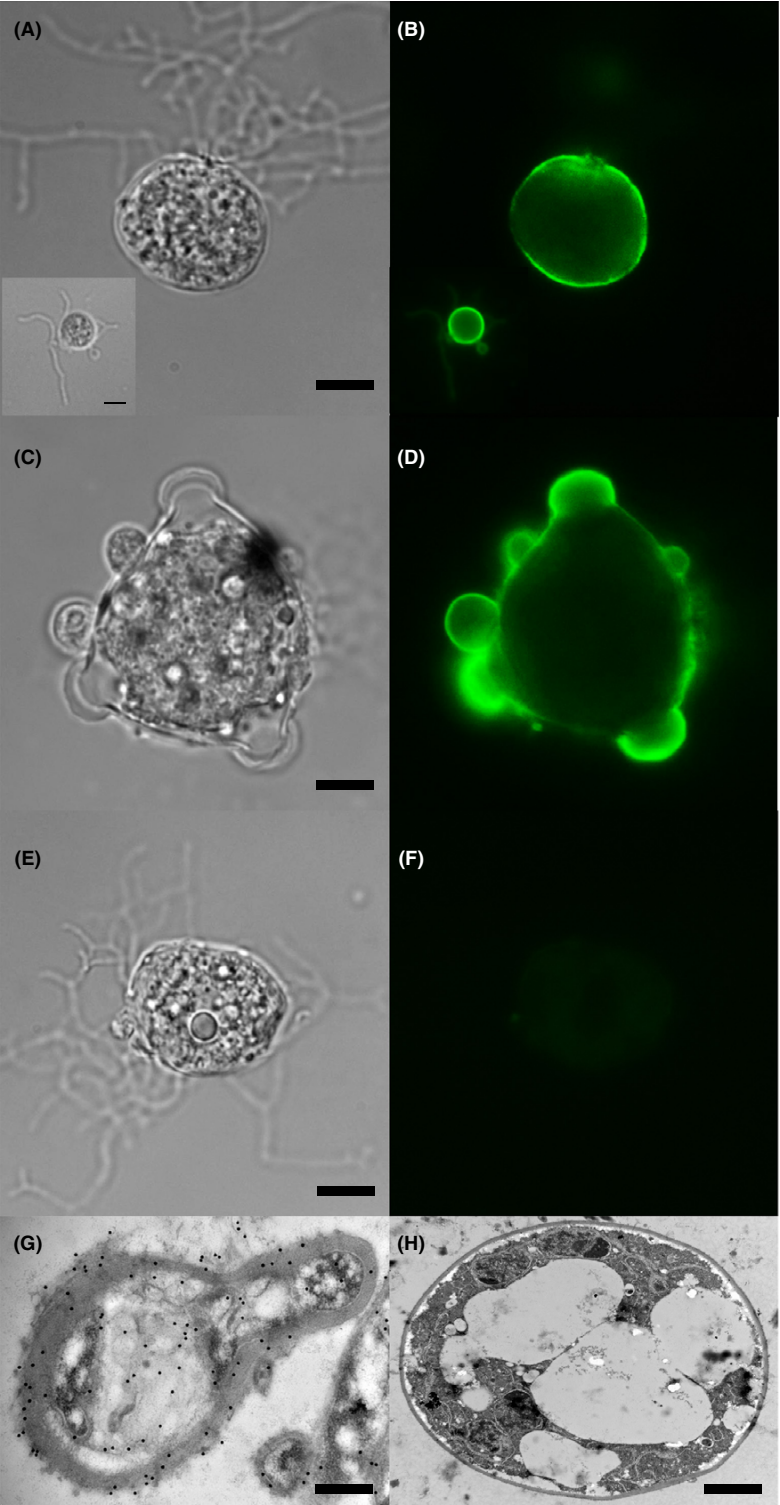
Cellular distribution of the 5C4 antigen. (A–F) Photomicrographs of *Bd*‐GPL JEL423 immunostained with 5C4 or TCM control and goat anti‐mouse polyvalent Ig fluorescein isothiocyanate (FITC) conjugate. (A) Brightfield image of sporangium with rhizoids (with brightfield image of encysted zoospore inset), probed with 5C4 followed by FITC conjugate. Scale bar = 4 μm (scale bar inset = 2.5 μm). (B) Same fields of view as A, but examined under epifluorescence. Note intense staining of the cell walls of the encysted zoospore and the sporangium, but lack of staining of rhizoids. (C) Brightfield image of mature zoosporangium with discharge papillae, probed with 5C4 followed by FITC conjugate, scale bar = 8.5 μm. (D) Same field of view as C but examined under epifluorescence. Note intense staining of the cell wall and papillae. (E) Brightfield image of sporangium with rhizoids, probed with TCM (negative control) followed by FITC conjugate, scale bar = 4 μm. (F) Same field of view as E but examined under epifluorescence. Note lack of staining, further demonstrating specific binding of 5C4 to surface antigen. (G and H) Immunogold labelling of sections of *Bd*‐GPL JEL423 cells. (G) Longitudinal section of cell incubated with 5C4 and anti‐mouse immunoglobulin 20 nm gold particles, showing antigen in cell wall, cytoplasm and in extracellular material surrounding the cell (scale bar = 0.65 μm). (H) Transverse section of cell incubated with TCM (negative control) and anti‐mouse immunoglobulin 20 nm gold particles, showing lack of staining by the secondary gold conjugate (scale bar = 3.5 μm).

Immunolocalization studies using IF showed that the 5C4 antigen was present on the surface of developing sporangia (Fig. 3A and B), on germlings (young sporangia) derived from encysted zoospores (Fig. 3A and B, inset) and on the surface of mature zoosporangia with discharge papillae (Fig. 3C and D), while immunogold electron microscopy (IEM) showed that the antigen was present in the cytoplasm, cell wall and extracellular material surrounding cells (Fig. 3G). No binding of 5C4 to rhizoids was evident in IF studies (Fig. 3A–F).

### Specificity of the LFA

Related and unrelated species of fungi that reacted with 5C4 in ELISA (*C. confervae*,* H. polyrhiza* and *Trichosporon* spp.) were tested for reactivity with 5C4 in the LFA format (Table S2). Only isolates of *Bd*,* Bsal* and *H. polyrhiza* gave positive LFA test results (test line and internal control line), with *C. confervae* and *Trichosporon* species giving negative results (single internal control line only) at similar concentrations of soluble antigens (250 μg protein mL^−1^) prepared from replicate slope cultures of the fungi. Consequently, while cross‐reactivity of 5C4 was found in the ELISA format with *C. confervae* and *Trichosporon* spp., cross‐reactivity with 5C4 was eliminated in the LFA format, making the LFA specific for *Bd*,* Bsal* and *H. polyrhiza*.

### LFA and qPCR detection of Bd infection in an animal model of chytridiomycosis

**Figure 4:**
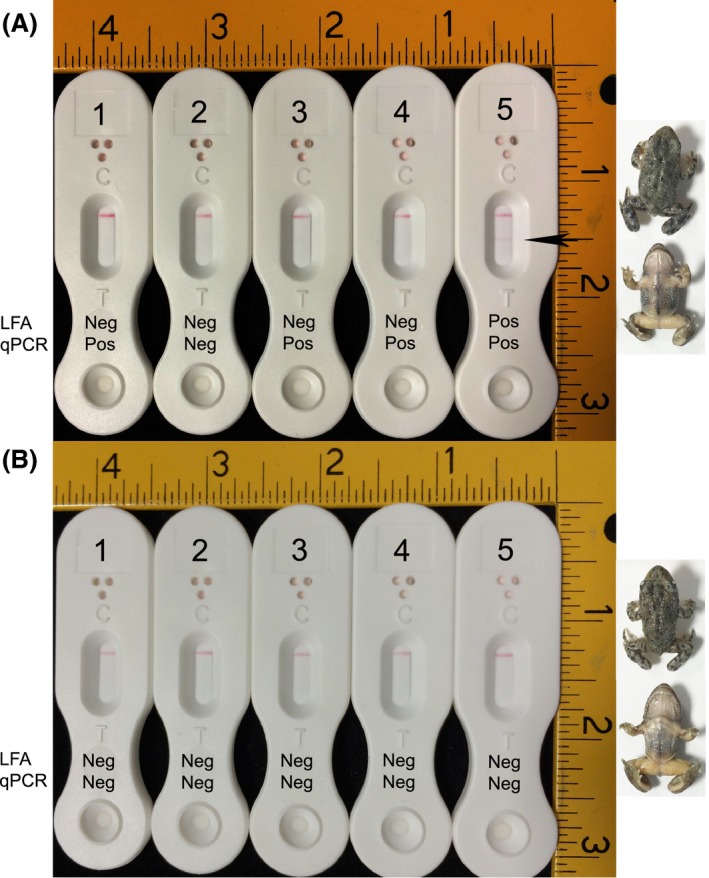
LFA and qPCR detection of *Bd* antigen and DNA in artificially infected animals. Juveniles of the Common Nurse toad (*Alytes obstetricans*) were exposed to zoospores of *Bd* (A) or to culture medium only (B), and presence of pathogen DNA or antigen determined after 23 days using qPCR or LFA tests of skin swabs. (A) qPCR and LFA test results (negative or positive) for each of the five replicate animals exposed to *Bd* zoospores. A positive (Pos) qPCR result equates to a GE value ≥ 0.1, while a positive LFA test result is indicated by the presence of two lines (test line (T) and internal control line (C)) and a negative result by the presence of a single internal control line (C) only. While four of five of the zoospore‐exposed animals were positive by qPCR at day 23 (animals 1, 3, 4 and 5), only a single animal (animal 5) was LFA positive (both control and test line (indicated by arrow) present after 15 min). Dorsal (upper) and ventral (lower) images of this qPCR‐ and LFA‐positive animal are shown to the right of A. (B) PCR and LFA test results for each of the five replicate control animals. All five animals were negative (Neg) by qPCR and LFA at day 23. Dorsal (upper) and ventral (lower) images of control animal 5 are shown to the right of B. Scale bars are in inches (1 inch = 2.54 cm), and images of LFA devices and animals are shown to scale.

Replicate juvenile common midwife toads (*Alytes obstetricans*) were exposed to *Bd* zoospores and, after 23 days, were tested for the presence of pathogen DNA and antigen in skin swabs using qPCR or the LFA respectively. All five replicate control animals (exposed to *Bd* culture medium only) were negative by both qPCR and LFA (Fig. 4B). Four of five of the *Bd*‐exposed animals were positive by qPCR, with genomic equivalent (GE) values of 0.950, 0.000, 4.901, 107.985 and 0.932 respectively (a GE value ≥ 0.1 indicating *Bd* infection). Only a single *Bd*‐exposed animal (replicate 5 with the lowest GE‐positive value of 0.932) was positive for *Bd* antigen in LFA tests (Fig. 4A).

### Immunodetection of 5C4 antigen in amphibian tissues naturally infected with Bd and Bsal

The ability of mAb 5C4 to detect its target antigen in naturally infected animals was determined through a double‐blind study using swabs of frozen archived tissue samples from animals previously confirmed as infected with *Bd* or *Bsal*, or non‐infected, by qPCR and/or histology (Table 4). In ELISA tests, 5C4 correctly identified 5 of 5 *Bd* qPCR‐positive tissue samples and 1 of 3 *Bsal* qPCR‐positive samples, but did not react with 26 of 26 qPCR‐negative samples. While the absorbance values for these *in vivo* ELISA tests were low compared with *in vitro* specificity tests using antigens from axenic cultures of *Bd* and *Bsal* (Fig. 1), the values were greater than the threshold absorbance value for test positivity (≥ 0.100). The low values were likely due to the small size of the tissue samples swabbed (feet, pelvices and skin fragments), which had been subjected to some freeze‐thawing over several years of storage.

**Table 4:** Results of qPCR, ELISA and LFA tests using swabs from amphibian foot, pelvic and skin samples

Sample number	Amphibian species	*Bd* qPCR^a^	*Bsal* qPCR^a^	Absorbance (450 nm)^b^	LFD result^d^
25	*Chioglossa lusitanica*	Negative	44.57	0.092 ± 0.006	+
6	*Litorea caerulea*	361.26	ND	0.111 ± 0.011	+
7	*Litorea caerulea*	320.45	ND	0.110 ± 0.009	+
11	*Litorea caerulea*	61.41	ND	0.114 ± 0.022	+
12	*Litorea caerulea*	3.75	ND	0.104 ± 0.004	+
10	*Litorea caerulea*	1.09	ND	0.100 ± 0.009	+
24	*Triturus pygmaeus*	Negative	3.56	0.133 ± 0.016	+
23	*Triturus pygmaeus*	Negative	3.75	0.077 ± 0.006	+
3^c^	*Litorea caerulea*	Negative	Negative	0.121 ± 0.002	+
17	*Agalychnis callidryas*	Negative	ND	0.074 ± 0.015	‐
8	*Bufo bufo*	Negative	ND	0.075 ± 0.002	‐
21	*Dendrobates auratus*	Negative	Negative	0.071 ± 0.008	‐
26	*Dendrobates auratus*	Negative	ND	0.078 ± 0.001	‐
18	*Dendrobates azureus*	Negative	ND	0.075 ± 0.011	‐
14	*Dendrobates leucomelas*	Negative	ND	0.074 ± 0.009	‐
15	*Dendrobates leucomelas*	Negative	ND	0.077 ± 0.007	‐
19	*Echinotriton andersoni*	Negative	ND	0.068 ± 0.003	‐
20	*Echinotriton andersoni*	Negative	ND	0.066 ± 0.001	‐
31	*Ichthyosaura alpestris*	Negative	Negative	0.072 ± 0.005	‐
32	*Ichthyosaura alpestris*	Negative	Negative	0.083 ± 0.004	‐
35	*Ichthyosaura alpestris*	Negative	Negative	0.073 ± 0.004	‐
27	*Lissotriton italicus*	Negative	Negative	0.078 ± 0.015	‐
4	*Pelobates cultripes*	Negative	ND	0.085 ± 0.004	‐
29	*Phyllomedusa hypochondrialis*	Negative	ND	0.069 ± 0.004	‐
30	*Phyllomedusa hypochondrialis*	Negative	ND	0.070 ± 0.007	‐
5	*Phyllobates terribilis*	Negative	ND	0.068 ± 0.004	‐
9	*Phyllobates terribilis*	Negative	ND	0.073 ± 0.006	‐
13	*Phyllobates terribilis*	Negative	ND	0.085 ± 0.014	‐
16	*Phyllobates terribilis*	Negative	ND	0.067 ± 0.006	‐
2	*Scaphiophryne marmorata*	Negative	ND	0.075 ± 0.007	‐
1	*Taricha torosa*	Negative	ND	0.086 ± 0.029	‐
22	*Theloderma stellatum*	Negative	ND	0.073 ± 0.004	‐
28	*Tylotriton shanjing*	Negative	Negative	0.088 ± 0.004	‐
33	*Tylotriton shanjing*	Negative	Negative	0.085 ± 0.005	‐
34	*Tylotriton shanjing*	Negative	Negative	0.073 ± 0.003	‐

a Total *Bd* or *Bsal* quantity, as determined by the Institute of Zoology by qPCR (Blooi *et al*., 2013); ND, not determined.

b Each value is the mean of three replicate samples. Threshold absorbance value for test positivity is ≥ 0.100.

c Sample 3 positive by histology for a chytrid‐like infection.

d + positive (test line and internal control line visible); ‐ negative (internal control line only) after 15 min.

**Figure 5:**
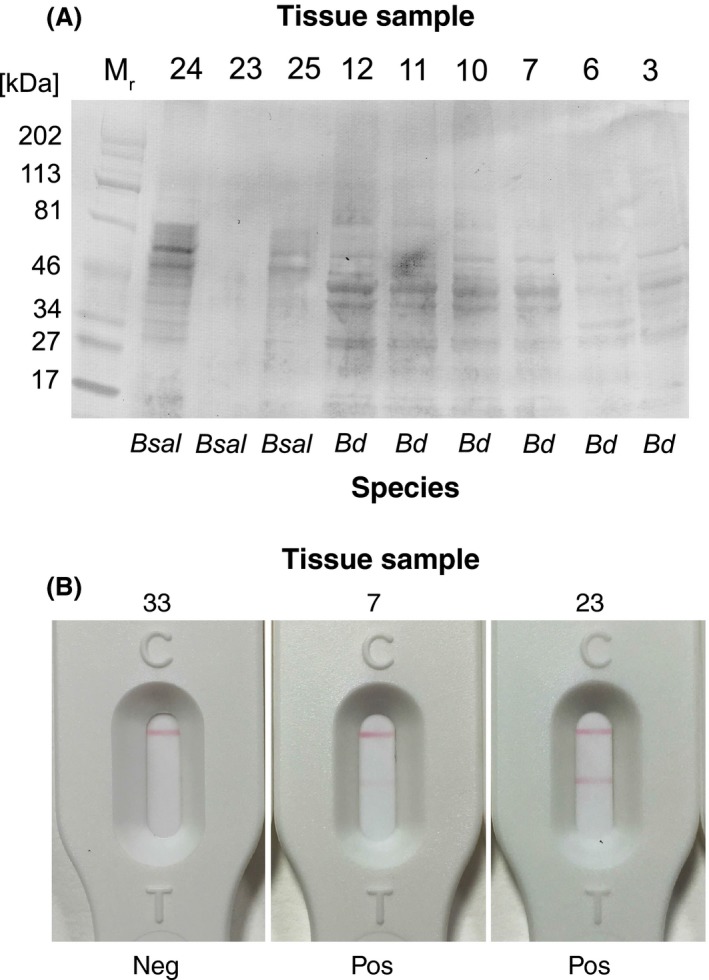
Immunodetection of 5C4 antigen in amphibian tissue samples. (A) Western immunoblot of soluble antigens in swabs of amphibian tissues naturally infected with *Bd* and *Bsal*. Soluble antigens present in swabs of foot, pelvic or skin fragments were subjected to denaturing SDS‐PAGE and transferred electrophoretically to a PVDF membrane. The membrane was probed with tissue culture supernatant of 5C4 followed by goat anti‐mouse IgM (μ‐chain‐specific) alkaline phosphatase conjugate and BCIP/NBT substrate. The antibody reacted with soluble antigens present in tissue swabs from frogs (samples 3, 6, 7, 10, 11 and 12) and newts and salamanders (samples 24 and 25) naturally infected with *Batrachochytrium dendrobatidis* (*Bd*) and *Batrachochytrium salamandrivorans* (*Bsal*) respectively. The 5C4 antibody was able to differentiate between *Bd* and *Bsal*, giving two distinct patterns of antigen binding. The 5C4‐negative sample 23 from a Southern Marbled newt (*Triturus pygmaeus*) was similarly negative in ELISA tests with 5C4, but was positive by *Bsal* qPCR and strongly positive with the 5C4 lateral‐flow assay (Table 4). The 5C4‐positive sample 3 from an Australian green tree frog (*Litoria caerulea*), which was negative by *Bd* qPCR (Table 4), was positive in ELISA and LFA tests and, in histology, this animal was shown to have a chytrid‐like infection. (B) Strong positive LFA test result for swab from tissue sample 23 (*Bsal* qPCR positive, Western blot negative and ELISA negative), weak positive LFA test result for sample 7 (*Bd* qPCR positive, Western blot positive and ELISA positive with 5C4) and negative LFA test result for sample 33 (negative by *Bd* qPCR, negative by *Bsal* qPCR and negative by Western blot and ELISA with 5C4).

In Western blotting studies (Fig. 5A) conducted with the same swab samples, 5C4 was able to discriminate between *Bd* (samples 3, 6, 7, 10, 11 and 12) and *Bsal* (lanes 24 and 25), producing distinct patterns of antigen binding for the two species. It was not able to detect antigen in *Bsal* sample 23, which was also negative with 5C4 in ELISA tests, but correctly identified sample 25 as *Bsal*, which was similarly negative for the 5C4 antigen in ELISA. Sample 3 was positive in ELISA tests with 5C4 (Table 4) and also produced a *Bd*‐indicative binding pattern in Western blots (Fig. 5A). This sample, while negative for *Bd* and *Bsal* by qPCR, was positive on histology for a chytrid‐like infection, although the identity of the infecting organism is unknown.

The LFA correctly identified *Bd* and *Bsal* in all eight of the qPCR‐positive amphibian samples and the single histology‐positive sample (Table 4). Eight of these nine positive samples gave weak positive LFA test results, while sample 23, negative by ELISA and Western blot, gave a strong positive LFA test result. The remaining 26 qPCR‐negative samples were also negative by LFA (Table 4). The LFA test results for samples 7 (weak positive), 23 (strong positive) and 33 (negative) are shown in Fig. 5B.

## Discussion

The primitive waterborne fungus *Bd* causes chytridiomycosis, an epizootic disease of amphibians. The global trade in amphibians has been implicated in the spread of the pathogen, and it has now been found on every continent where amphibians occur (Fisher *et al*., 2009; Schloegel *et al*., 2012; Olson *et al*., 2013). More recently, the related and highly pathogenic chytrid *Bsal* has emerged as the aetiological agent responsible for the extirpation of fire salamander populations in the Netherlands (Martel *et al*., 2013).

The World Organisation for Animal Health (OIE, 2016) has declared *Bd* as a notifiable pathogen, recommending that all imported and exported amphibians be screened for its presence. Despite this, current screening protocols are laborious and time‐consuming, with potentially infectious samples needing to be sent to a diagnostic laboratory appropriately equipped for analysis using PCR (Annis *et al*., 2004; Boyle *et al*., 2004; Kriger *et al*., 2006). Histological examination can be used as an alternative to PCR, but this method is invasive and requires trained personnel to identify fungal structures in tissue samples (Berger *et al*., 1999b), meaning that it is best suited to post‐mortem identification of infected animals. The paucity of quick and accurate detection methods means there is a pressing need for a more rapid, cheap, portable and user‐friendly diagnostic assay that can be used to monitor pathogen presence.

This study describes the development of a highly specific monoclonal antibody (mAb), 5C4, and its incorporation into a simple, single‐step, LFA for the rapid detection of *Bd* and *Bsal* diagnostic antigen. While a LFA has been developed for the diagnosis of amphibian ranavirus (Kim *et al*., 2015), this is the first time that a mAb‐based LFA has been developed for tracking chytrid pathogens of amphibians.

Monoclonal antibody 5C4, which binds to a carbohydrate epitope present on an extracellular, heat‐stable, ~27 to ~220 kDa glycoprotein antigen located on zoospores, germlings, sporangia and zoosporangia of *Bd*, is highly specific, recognizing geographically distinct members of the hypervirulent global pandemic lineage of *Bd* (*Bd*‐GPL), and isolates of the related chytrid *Bsal* pathogenic to salamanders and newts. The mAb also reacts with the newly described non‐pathogenic chytrid *H. polyrhiza* isolated from lake water and which is grouped in molecular hypotheses with *Bd* (Longcore *et al*., 2011), but does not react with other members of the *Rhizophydiales*, namely *Entophlyctis*,* Rhizophlyctis* and *Rhizophydium* spp. It does not cross‐react with a wide range of unrelated fungi and oomycetes that reside in terrestrial and aquatic environments occupied by amphibians. Nevertheless, cross‐reactivity of 5C4 was shown in ELISA with the related chytrid fungus *C. confervae* and with certain species of the unrelated fungal genus *Trichosporon*. SSU rDNA sequencing shows that *Bd*,* Bsal* and *C. confervae* all form a single phylogenetic clade (Berger *et al*., 1998; Martel *et al*., 2013), and so cross‐reactivity with this closely related fungus is not unexpected. As an environmental saprotroph, it is not known to cause disease in vertebrates and has not been reported in association with amphibians (Canter and Lund, 1962; Gauriloff and Fuller, 1979). *Trichosporon* spp. are basidiomycete yeast‐like fungi that are found in a diverse range of habitats, including soil, rivers and lakes (Colombo *et al*., 2011), with limited evidence for their association with the skin of frogs (Mok and Morato de Carvalho, 1985; Sammon *et al*., 2010). While 5C4 was reactive with *H. polyrhiza* in both the ELISA and LFA formats, cross‐reactivity of 5C4 was not found with *Chytridium* and *Trichosporon* spp. in the LFA format. The reasons for this are not immediately apparent, but a possible factor for the elimination of 5C4 cross‐reactivity in the LFA is the configuration of the target antigen in this immunoassay format. In the ELISA, where the antigen is immobilized to a solid phase, all of the carbohydrate binding sites may be displayed for antibody binding, whereas in the LFA (antigen sandwich), only those on the surface of the antigen are displayed. The binding of carbohydrate‐specific IgM antibodies to repeat epitopes is well documented, so too ‘context‐dependant recognition’ and the ability of multivalent IgM molecules to differentiate between many copies of a carbohydrate antigen and just a few (Haji‐Ghassemi *et al*., 2015). Notwithstanding this, the improved specificity of 5C4 in the LFA format, compared with the ELISA, means that the assay is highly specific for detection of *Bd*,* Bsal* and *H. polyrhiza*.

The accuracy of 5C4 in detecting its target antigen in amphibian skin samples was investigated using (i) experimental animals artificially infected with *Bd* and (ii) using tissue samples recovered from animals naturally infected with *Bd* and *Bsal*. Paradoxically, the LFA proved less able to detect the 5C4 antigen in swabs from juveniles of the toad *A. obstetricans* artificially infected under controlled conditions, compared with the naturally infected tissue samples. However, as the artificially infected animals were only subjected to three rounds of inoculum over a relatively short experimental timeframe (23 days), the majority of zoosporangia would be immature and buried in the stratum corneum, which would likely impact the ability of the swabbing process to access antigen for LFA detection. It is also possible that in the infection study conducted here, the LFA detected the zoospore inoculum on the skin surface itself rather than infection *per se*. Indeed, we have shown here, in Western blotting studies, detection of zoospore‐associated antigen by 5C4. Furthermore, binding of 5C4 to *Bd* zoospores on the skin surface during the infection of zebrafish larvae has also recently been demonstrated using immunofluorescence (IF) microscopy (Liew *et al*., 2016).

Despite the discrepancies between qPCR and LFA using artificially infected animals, there was good concordance between the two detection methods in tests of naturally infected tissues. Detection of the 5C4 antigen across a range of amphibian species was investigated using three different immunoassay formats, namely ELISA, Western blotting and the LFA. In a double‐blind study of swab samples from naturally infected animals, 5C4 was able to differentiate *Bd* from *Bsal* in Western blots, providing unique antigen binding profiles for the two *Batrachochytrium* species. Serological differentiation of related species of human and plant pathogenic fungi in Western blots using experimentally induced rabbit antisera has been reported previously (Moragues *et al*., 2001; Bulajić *et al*., 2007), but this is the first demonstration of the ability of a mouse mAb to visually discriminate between related species of pathogenic fungi infecting animal tissues. Unlike the ELISA that detected the 5C4 antigen in 88% (seven of eight) of the *Bd* or *Bsal* qPCR‐positive tissue samples, the LFA was positive with 100% (eight of eight) of the qPCR‐positive samples. In addition, both the LFA and ELISA were positive for a single qPCR‐negative sample that was positive in histology for a chytrid‐like infection.

Taken together, these results demonstrate that the portable LFA has the potential to be used as a qualitative front‐line test to rapidly detect *Bd* or *Bsal* antigen in naturally infected frogs, newts and salamanders. Laboratory‐based PCR could then be used to confirm their presence and to differentiate the infecting species. To this end, field‐testing of the LFA will be undertaken to determine its efficacy as a rapid adjunct test for environmental detection of the pathogens.

## Experimental procedures

### Ethics statement

All animal work relating to hybridoma production was conducted under a UK Home Office Project Licence and was reviewed by the institution's Animal Welfare Ethical Review Board for approval. It was carried out in accordance with The Animals (Scientific Procedures) Act of 1986 Directive 2010/63/EU and followed all of the Codes of Practice which reinforce this law, including all elements of housing, care and euthanasia. Amphibian infection studies were carried out under a UK Home Office Licence held by M.C. Fisher.

### Fungal culture

We used members of four known *Bd* lineages to ensure that all were recognized by the mAb: lineage *Bd*‐GPL JEL423 (Panama) and *Bd*‐GPL 08MG02 (South Africa); lineage *Bd*‐ASIA KBOOR317 (Korea); lineage *Bd*‐CAPE SA4c (South Africa) and *Bd*‐CAPE TF5a1 (Mallorca); and lineage *Bd*‐SWISS 0739 (Switzerland). The chytrid fungus *H. polyrhiza* JEL142 was purchased from the culture collection of J. Longcore. The *Bd*‐GPL isolate JEL423, all other isolates of *Bd* and *Bsal* (Table S1) and *H. polyrhiza* were cultured in tryptone–gelatin hydrolysate–lactose broth (TGhL; tryptone 16 g L^−1^, gelatin hydrolysate 4 g L^−1^, lactose 2 g L^−1^) or on peptone–malt extract–glucose agar (ARCH; peptone 2 g L^−1^, malt extract 3 g L^−1^, glucose 8 g L^−1^, agar 8 g L^−1^) at 23°C under a 16 h photoperiod of fluorescent light. The chytrid fungi *Chytridium*,* Entophlyctis*,* Phlyctochytrium*,* Rhizophlyctis* and *Rhizophydium* were also cultured on ARCH medium under similar conditions. *Allomyces* species were cultured on oatmeal agar (OA: O3506; Sigma, Poole, Dorset, United Kingdom), *Pythium* and *Saprolegnia* species on corn meal agar (CMA: C1176; Sigma) and all three were grown at 26°C under a 16 h photoperiod of fluorescent light. All other fungi were cultured on glucose–peptone–yeast extract agar (glucose 40 g L^−1^, bacteriological peptone 5 g L^−1^, yeast extract 5 g L^−1^, agar 15 g L^−1^), malt extract agar (M6907; Sigma), yeast malt agar (Y3127; Sigma), potato dextrose agar (P2182; Sigma) or Sabouraud dextrose agar (SDB: S3306; Sigma and agar 20 g L^−1^) as described previously (Davies and Thornton, 2014). All media were sterilized by autoclaving at 121°C for 15 min.

### Preparation of immunogen, immunization regime and production of hybridoma cell lines

Replicate 50 mL tissue culture flasks (TCF‐012‐050; Jet Biofil, Madrid, Spain) containing 10 mL TGhL were inoculated with 10^3^ sporangia mL^−1^ of *Bd*‐GPL isolate JEL423. After 4 days growth at 23°C, adherent cells (encysted zoospores and zoosporangia) were harvested using a sterile cell scraper (C5981; Sigma) and, along with the medium containing motile zoospores, were snap‐frozen in liquid N_2_, lyophilized for 5 days and stored at −20°C prior to use. Immunogen was prepared by suspending 2 mg lyophilized material in 1 mL phosphate buffer saline (PBS; 137 mM NaCl, 2.7 mM KCl, 8 mM Na_2_HPO_4_, 1.5 mM KH_2_PO_4_, pH 7.2) and 6‐week‐old BALB/c mice were each given four consecutive intraperitoneal injections (300 μL per injection) of immunogen at 2‐week intervals. A single booster injection was given 5 days before fusion, and hybridoma cells were produced as described previously (Thornton, 2001). For zoospore isolation, the method of Myers *et al*. (2012) was used, with modification. JEL423 was cultured in TGhL as described and, after 4 days growth at 23°C, the medium containing zoospores was aspirated with a pipette and passed through a sterile coffee filter to remove sporangia. The zoospore suspension was centrifuged at 14 462 *g* for 5 min, the supernatant removed and the pelleted cells re‐suspended in sterile MQ‐H_2_O. The cells were washed by repeated centrifugation and re‐suspension in MQ‐H_2_O three times, and the final zoospore suspension then snap‐frozen in liquid N_2_ and lyophilized as described.

### Screening of hybridomas by enzyme‐linked immunosorbent assay

Antibody‐producing hybridomas were first identified in ELISA using solubilized antigens from the *Bd*‐GPL JEL423 immunogen. Solubilized antigens were prepared by centrifugation of the immunogen at 14 462 *g* for 5 min, and 50 μL volumes of the supernatant containing soluble antigens used to coat the wells of microtitre plates (Nunc Maxisorp; Fisher Scientific UK Ltd., Loughborough, Leicestershire, United Kingdom), by overnight incubation at 4°C in sealed plastic bags. Positive cell lines were subsequently tested for mAb specificities against surface washings containing soluble antigens, prepared from replicate slopes of fungi (Table S1) as described in Thornton (2001). Protein concentrations, determined spectrophotometrically at 280 nm (Nanodrop; Agilent Technologies, Stockport, Chesire, United Kingdom), were adjusted to 250 μg mL^−1^, and 50 μL volumes were used to coat the wells of microtitre plates, which were incubated overnight at 4°C as described. Antigen‐coated plates were washed three times with PBST (PBS containing 0.05% (v/v) Tween‐20), once with PBS and once with dH_2_O before being air‐dried at 23°C in a laminar flow hood. The plates were sealed in plastic bags and stored at 4°C in preparation for screening of hybridoma supernatants by ELISA.

For ELISA, wells containing immobilized antigens were blocked for 15 min with 100 μL of PBS containing 1.0% (w/v) bovine serum albumin (BSA: A2153; Sigma). After a 5‐min rinse with PBS, wells were incubated with 50 μL of hybridoma tissue culture supernatant (TCS) for 1 h, after which they were washed three times, for 5 min each, with PBST. Goat anti‐mouse polyvalent immunoglobulin (classes IgG, IgA and IgM) peroxidase conjugate (A0412; Sigma), diluted 1:1000 in PBST containing 0.5% BSA, was added to the wells and incubated for a further hour. The plates were washed with PBST as described, given a final 5 min wash with PBS, and bound antibody visualized by incubating wells with tetramethyl benzidine (TMB: T2885; Sigma) substrate solution for 30 min, after which reactions were stopped by the addition of 3 M H_2_SO_4_. Absorbance values were determined at 450 nm using a microplate reader (Tecan GENios, Reading, Berkshire, United Kingdom). Control wells were incubated with tissue culture medium (TCM) containing 10% fetal bovine serum (FBS; Labtech International Ltd., Uckfield, East Sussex, United Kingdom) only. All incubation steps were performed at 23°C in sealed plastic bags. The threshold for detection of the antigen in ELISA was determined from control means (2 × TCM absorbance values) (Sutula *et al*., 1986). These values were consistently in the range 0.050–0.100. Consequently, absorbance values ≥ 0.100 were considered as positive for the detection of antigen.

### Determination of Ig subclass and subcloning procedure

The Ig class of mAbs was determined using plate‐trapped antigen ELISA. Wells of microtitre plates coated with soluble antigens from the *Bd*‐GPL JEL423 immunogen were incubated successively with TCS for 1 h, followed by goat anti‐mouse IgG1, IgG2a, IgG2b, IgG3, IgM or IgA‐specific antiserum (ISO‐2; Sigma) diluted 1:3000 in PBST for 30 min and rabbit anti‐goat peroxidase conjugate (A5420; Sigma) diluted 1:5000 for a further 30 min. Bound antibody was visualized with TMB substrate as described. Hybridoma cell lines were subcloned three times by limiting dilution, and cell lines were grown in bulk in a non‐selective medium, preserved by slowly freezing in FBS/dimethyl sulfoxide (92:8 v/v), and stored in liquid N_2_.

### Epitope characterization by heat, chemical and enzymatic modification

Heat stability of the 5C4 antigen was investigated by placing solubilized antigen from *Bd*‐GPL JEL423 immunogen in a boiling water bath. At 10 min intervals over a 60 min period, 1 mL samples were removed, cooled and centrifuged at 14 462 *g* for 5 min. Fifty microlitre volumes of supernatants were immobilized to the wells of microtitre plates for assay by ELISA as described. For periodate oxidation, microtitre wells coated with solubilized antigen from the *Bd*‐GPL JEL423 immunogen were incubated with 50 μL of sodium metaperiodate solution (20 mM NaIO_4_ in 50 mM sodium acetate buffer, pH 4.5) or acetate buffer only (control) for 16, 4, 3, 2, 1 or 0 h at 4°C in sealed plastic bags. Plates were given four 3 min PBS washes before processing by ELISA as described. For protease digestions, microtitre wells containing immobilized antigens were incubated with 50 μL of a 0.9 mg mL^−1^ solution of pronase (protease XIV; Sigma), trypsin solution (1 mg mL^−1^ in MQ‐H_2_O) or PBS and MQ‐H_2_O only (controls) for 4 h at 37°C or 4°C. Plates were given four 3 min rinses with PBS and then assayed by ELISA with 5C4 as described.

### Gel electrophoresis and Western blotting

For sodium dodecyl sulphate‐polyacrylamide gel electrophoresis (SDS‐PAGE), *Bd*‐GPL JEL423 immunogen or washed zoospore preparation was reconstituted in Laemmli buffer (Laemmli, 1970) and denatured by heating at 100°C for 10 min. SDS‐PAGE was carried out using 4–20% (w/v) gradient polyacrylamide gels (161‐1159; Bio‐Rad Laboratories Ltd., Hemel Hempstead, Hertfordshire, United Kingdom) under denaturing conditions. Proteins were separated electrophoretically at 23°C (165 V), and prestained broad‐range markers (161‐0318; Bio‐Rad) were used for molecular weight determinations. For Westerns, separated proteins were transferred electrophoretically to a PVDF membrane (162‐0175; Bio‐Rad) for 2 h at 75 V. To further study the sensitivity of the 5C4 antigen to periodate oxidation, membranes were incubated for 24 h at 4°C in acetate or periodate solutions prepared as described and, after washing three times with PBS, were blocked for 16 h at 4°C with PBS containing 1% (w/v) BSA. The blocked membranes were incubated with 5C4 TCS diluted 1:2 (v/v) with PBS containing 0.5% BSA (PBSA) for 2 h at 23°C. After washing three times with PBS, the membrane was incubated for 1 h with goat anti‐mouse IgM (μ‐chain‐specific) alkaline phosphatase conjugate (A9688; Sigma), diluted 1:15 000 in PBSA. The membrane was washed three times with PBS, once with PBST and bound antibody visualized by incubation in BCIP/NBT substrate solution (Thornton, 2008). Reactions were stopped by immersion in dH_2_O and air‐dried between sheets of Whatman filter paper.

### Spatio‐temporal localization of antigen by ELISA, IF and immunogold electron microscopy

To investigate extracellular production of the 5C4 antigen, 4‐day‐old TGhL cultures of *Bd*‐GPL JEL423 were harvested as described and the cells were pelleted by centrifugation at 14 462 *g* for 10 min. Extracellular antigens generated during the 4 day culture period were removed by washing the cells three times with fresh TGhL medium by repeated centrifugation and re‐suspension. The cells were finally re‐suspended in TGhL medium, and replicate 75 cm^2^ tissue culture flasks containing 10 mL TGhl were inoculated with 10^3^ washed sporangia mL^−1^. The newly generated cultures were incubated at 23°C under a 16 h fluorescent light regime and, at 24 h intervals over a 7 day period, 100 μL samples were removed from each flask, centrifuged at 14 462 *g* for 5 minutes to pellet cells and 50 μL volumes of supernatants immobilized to the wells of microtitre plates for assay by ELISA as described.

To study the cellular distribution of the 5C4 antigen, IF and immunogold electron microscopy of cells were used. For IF, 200 μL volumes of TGhL medium containing washed sporangia were placed on the surface of sterilized glass slides and were incubated for 24 h at 23°C in a moist chamber. Slides were allowed to air‐dry at 23°C in a laminar flow cabinet before the cells were fixed to the slides as described in Thornton (2001). Fixed cells were incubated with 5C4 TCS or TCM only (negative control) for 1 h at room temperature, followed by three washes with PBS. Samples were then incubated for 30 min at 23°C with goat anti‐mouse polyvalent fluorescein isothiocyanate (FITC) conjugate (F1010; Sigma) diluted 1 in 40 in PBS. Slides were given three 5 min washes with PBS and mounted in PBS–glycerol mounting medium (F4680; Sigma) before overlaying with coverslips. All incubation steps were performed at 23°C in a humid environment to prevent evaporation, and slides were stored in the dark, at 4°C, prior to examination using an epifluorescence microscope (Olympus IX81) fitted with 495 nm (excitation) and 518 nm (emission) filters for FITC.

For IEM, the method described in Thornton and Talbot (2006) was used. Washed cells were embedded in LR White resin (Agar Scientific, Stansted, Essex, United Kingdom) and ultra‐thin sections prepared for immunolabelling. Sections immobilized to nickel grids were blocked by immersion in PBST containing 1% (w/v) BSA (PBST‐BSA) which had been sterile‐filtered through a 0.2 μm filter. The grids were washed three times (3 min each) in sterile‐filtered PBST and then incubated in 5C4 TCS or TCM only (negative control) for 1 h. After four washes (3 min each) with sterile‐filtered PBST, the grids were incubated for a further hour in PBST‐BSA containing a 1:20 (v/v) dilution of goat anti‐mouse 20 nm gold conjugate (EM.GAF20; BBI Solutions, Cardiff, Wales, United Kingdom). The grids were washed four times (3 min each) in sterile‐filtered PBST and then placed on Whatman filter paper to dry. Dried grids were then incubated for 20 min in 2% (w/v) uranyl acetate solution followed by 2% (w/v) lead citrate solution for 4 min. Working volumes were 100 μL, and incubation and washing steps were carried out at 23°C. Immunostained samples were examined using a Jeol JEM 1400 transmission electron microscope fitted with a Gatan ES 100W CCD camera.

### Configuration of the LFA and determination of specificity

The LFA consisted of a Kenosha backing card, CP7 conjugate pad, A205 sample pad and Millipore HF135 polyester‐backed nitrocellulose membrane (GE Healthcare Life Sciences, Little Chalfont, Buckinghampshire, United Kingdom). Monoclonal antibody 5C4 was purified using T‐Gel™ (44916; ThermoFisher Scientific, Warrington, United Kingdom), conjugated to 40 nm diameter gold particles, sprayed on to the release pad at OD8 and then air‐dried for 16 h at 23°C and 20% relative humidity. The test line antibody consisted of T‐Gel purified 5C4 at 0.75 mg protein mL^−1^, while a commercial goat anti‐mouse IgM μ‐chain‐specific immunoglobulin (115‐005‐020; Jackson ImmunoResearch Laboratories, Newmarket, Cambridgeshire, United Kingdom) at a concentration of 0.1 mg mL^−1^ acted as the internal control line. Membranes were housed in Vision Housing with Single Port V.3 bases and lids. For assay using the LFA, 100 μL of sample was applied to the release port of the device and, after 15 min, the results were recorded as positive for the presence of the antigen (two lines) or negative (a single internal control line only). For specificity tests, surface washings containing soluble antigens were prepared from slope cultures of fungi (Table S2), adjusted to 250 μg protein mL^−1^ and LFA results recorded as described.

### LFA and qPCR detection of Bd antigen and DNA in an animal model of chytridiomycosis

Experimental animals (juvenile *A. obstetricans*) were raised from tadpoles collected from the Western Pyrenees under licence from the French Pyrenean National Park. Animals were housed individually in 1 L plastic boxes with tissue paper soaked in aged tap water and fed *ad libitum* with live crickets. Five replicate experimental animals were exposed overnight to three doses of active *Bd* zoospores grown in liquid mTGhL medium, while control animals were exposed to uninoculated mTGhL only. Experimental animals were exposed to zoospores on day 1 (10 000 zoospores), day 5 (10 000 zoospores) and day 9 (30 000 zoospores) with *Bd*‐GPL isolate IT1 (passage 14), collected in Switzerland in 2011 from an infected *A. obstetricans*. The 23‐day experiment was conducted in a climate‐controlled room kept at 18°C and with a 12/12 h day/night light regime.

On day 23, all animals were tested for *Bd* antigen or DNA using qPCR for DNA and the LFA for antigen. Individual animals were swabbed using sterile MW100 cotton swabs and DNA extracted using the bead‐beating protocol outlined previously (Boyle *et al*., 2004). DNA extractions were diluted 1/10 before being used as templates for qPCR amplification (Boyle *et al*., 2004). All PCR were performed in duplicate, and with *Bd* GE standards of 100, 10, 1 and 0.1 GE. Samples generating GE estimates of 0.1 GE or greater were scored as positive. For LFA tests, sterile cotton swabs (Technical Service Consultants, Heywood, Lancashire, United Kingdom) were dipped in sterile water, the animals were wiped thoroughly and the tips of the swabs placed into 1 mL of sterile water containing 0.1% sodium azide. The samples were vortexed briefly, 50 μL volumes were mixed 1:1 (v/v) with PBST, and the combined 100 μL added to the devices and test results determined as described.

### Immunodetection of 5C4 antigen in amphibian tissues naturally infected with Bd and Bsal

Thirty‐five amphibian foot, pelvic or skin fragment samples from the Wildfowl & Wetlands Trust frozen archive were tested in ELISA, LFA and Western blot for the presence of the 5C4 antigen. Samples were thawed at 4°C overnight, before swabs were taken. Sterile cotton swabs (Technical Service Consultants) were dipped in sterile water, the tissue samples wiped thoroughly and the tips of the swabs placed into 1 mL of sterile water containing 0.1% (w/v) sodium azide. The samples were transported to the laboratory on ice, vortexed briefly and 50 μL volumes containing soluble antigen used to coat the well of microtitre plates for ELISA with 5C4 as described. For LFA tests, 50 μL volumes of vortexed samples were mixed 1:1 (v/v) with PBST and the combined 100 μL added to the devices and test results recorded as described. For Western blots, soluble antigens were denatured by heating in Laemmli buffer and, following SDS‐PAGE, were transferred to PVDF membrane and processed with 5C4 as described.

### Statistical analysis

Numerical data were analysed using the statistical programme Minitab (Minitab 16; Minitab^®^, Coventry, UK). Analysis of variance (ANOVA) was used to compare means, and post hoc Tukey–Kramer analysis was then performed to determine statistical significance.

## Conflict of Interest

We declare that none of the authors involved in writing this article have any conflict of interests with respect to its contents.

## Supporting information


**Table S1.** Fungi and oomycetes used in this study.

**Table S2.** Lateral‐flow assay test results for selected ELISA‐positive and ELISA‐negative fungi.
