# Supplementary material for: Tracking the amphibian pathogens Batrachochytrium dendrobatidis and Batrachochytrium salamandrivorans using a highly specific monoclonal antibody and lateral‐flow technology
Source: Microb Biotechnol. 2016 Dec 19;10(2):381–94. doi: 10.1111/1751-7915.12464 (PMC5328824; doi:10.1111/1751-7915.12464)
Supplement: Supplementary file 1 — Table S1. Fungi and oomycetes used in this study. [file MBT2-10-381-s001.docx]

|  | Table S1. Fungi and oomycetes used in this study. | | |
| --- | --- | --- | --- |
|  | **Organism** | **Isolate no.** | **Source^a^** |
|  |  |  |  |
| Chytridiomycota | *Batrachochytrium dendrobatidis* | GPL 08MG02 | MCF |
|  | *Batrachochytrium dendrobatidis* | GPL JEL423 | MCF |
|  | *Batrachochytrium dendrobatidis* | GPL KBOOR317 | MCF |
|  | *Batrachochytrium dendrobatidis* | CAPE SA4c | MCF |
|  | *Batrachochytrium dendrobatidis* | SWISS 0739 | MCF |
|  | *Batrachochytrium dendrobatidis* | CAPE TF5a1 | MCF |
|  | *Batrachochytrium salamandrivorans* | 135744 | CBS |
|  | *Batrachochytrium salamandrivorans* | UK LFRC1 | MCF |
|  | *Allomyces arbusculus* | 101.33 | CBS |
|  | *Allomyces arbusculus* | 104.36 | CBS |
|  | *Allomyces javanicus* | 600.78 | CBS |
|  | *Allomyces macrogynus* | 221.89 | CBS |
|  | *Allomyces moniliformis* | 104.67 | CBS |
|  | *Allomyces neomoniliformis* | 105.52 | CBS |
|  | *Chytridium confervae* | 675.73 | CBS |
|  | *Entophlyctis confervae-glomeratae* | 663.77 | CBS |
|  | *Homolaphlyctis polyrhiza* | JEL142 | JL |
|  | *Phlyctochytrium californicum* | 667.73 | CBS |
|  | *Phlyctochytrium reinboldtiae* | 669.73 | CBS |
|  | *Rhizophlyctis rosea* | 124.41 | CBS |
|  | *Rhizophydium sphaerocarpum-spirogyrae* | 777.73 | CBS |
|  |  |  |  |
| Oomycota | *Pythium insidiosum* | 101039 | CBS |
|  | *Pythium tracheiphilum* | 323.65 | CBS |
|  | *Saprolegnia diclina* | 113343 | CBS |
|  | *Saprolegnia ferax* | 283.38 | CBS |
|  | *Saprolegnia parasitica* | 113187 | CBS |
|  |  |  |  |
| Ascomycota | *Alternaria infectoria* | 137.90 | CBS |
|  | *Aspergillus flavus* | 91856iii | IMI |
|  | *Aspergillus fumugatus* | AF293 | CRT |
|  | *Aspergillus niger* | 702.40 | CBS |
|  | *Aspergillus restrictus* | 116.50 | CBS |
|  | *Aspergillus terreus* | 601.65 | CBS |
|  | *Aspergillus wentii* | 121.32 | CBS |
|  | *Botrytis cinerea* | R2 | CRT |
|  | *Candida albicans* | 90028 | ATCC |
|  | *Candida albicans* | NGY152 | CRT |
|  | *Candida albicans* | SC5132 | CRT |
|  | *Candida dubliniensis* var. *dubliniensis* | 8500 | CBS |
|  | *Candida guilliermondii* | B9-1 (KT876707) | CRT |
|  | *Candida intermedia* | C4-2 (KT876709) | CRT |
|  | *Candida lusitaniae* | C1-1 (KT876708) | CRT |
|  | *Candida orthopsilosis* | X6-4 (KT876504) | CRT |
|  | *Candida palmioleophila* | H3-4 (KT876573) | CRT |
|  | *Candida parapsilosis* var. *parapsilosis* | 8536 | CBS |
| Ascomycota | *Candida parapsilosis* | X9-2 (KT876496) | CRT |
|  | *Candida pseudotropicals* | 3234 | NCPF |
|  | *Candida sake* | 3860 | NCPF |
|  | *Candida tropicalis* var. *tropicalis* | 1920 | CBS |
|  | *Candida xylopsoci* | 6037 | CBS |
|  | *Chaetosartorya chrysella* | 4722.65 | CBS |
|  | *Corynespora cassiicola* | 296.80 | CBS |
|  | *Exophiala castellani* | G6-2 (KT876528) | CRT |
|  | *Exophiala dermatitidis* | P1-2 (KT876582) | CRT |
|  | *Exophiala dermatitidis* | X9-4 (KT876503) | CRT |
|  | *Exophiala pisciphila* | X9-7 (KT876502) | CRT |
|  | *Exophiala phaeomuriformis* | R9-3 (KT876555) | CRT |
|  | *Exophiala pisciphila* | A-10 (KT876616) | CRT |
|  | *Fusarium oxysporum* var. *lycopersici* | 167.30 | CBS |
|  | *Fusarium solani* | 224.34 | CBS |
|  | *Galactomyces candidum* | 113.23 | CBS |
|  | *Neosartoya ficheri* var. *ficheri* | 681.71 | CBS |
|  | *Penicillium cyclopium* | 123.14 | CBS |
|  | *Penicillium islandicum* | 338.48 | CBS |
|  | *Pichia norvegensis* | 6564 | CBS |
|  | *Scedosporium apiospermum* | 117467 | CBS |
|  | *Scedosporium aurantiacum* | 121926 | CBS |
|  | *Trichoderma hamatum* | GD12 | CRT |
|  | *Verticillium albo-atrum* | 312.91 | CBS |
|  |  |  |  |
| Basidiomycota | *Cryptococcus neoformans* var. *neoformans* | 7779 | CBS |
|  | *Cryptococcus neoformans* Serotype D | 5728 | CBS |
|  | *Cryptococcus saitoi* | 1975 | CBS |
|  | *Cystobasidium slooffiae* | A4-3 (KT876704) | CRT |
|  | *Filobasidiella bacillispora* | 10865 | CBS |
|  | *Magnusiomyces capitatus* | 207.83 | CBS |
|  | *Rhizoctonia solani* | 323.84 | CBS |
|  | *Rhodosporidium toruloides* | 6016 | CBS |
|  | *Rhodotorula glutinus* | H3-5 (KT876598) | CRT |
|  | *Rhodotorula mucilaginosa* | X5-3 (KT876501) | CRT |
|  | *Sporidiobolus salmonicolor* | 6781 | CBS |
|  | *Sporidiobolus salmonicolor* | 6832 | CBS |
|  | *Trichosporon asahii* | 2479 | CBS |
|  | *Trichosporon asahii* var. *asahii* | 8973 | CBS |
|  | *Trichosporon asteroides* | 2481 | CBS |
|  | *Trichosporon asteroides* | 6183 | CBS |
|  | *Trichosporon asteroides* | 7623 | CBS |
| Basidiomycota | *Trichosporon asteroides* | 7624 | CBS |
|  | *Trichosporon dermatis* | 2043 | CBS |
|  | *Trichosporon domesticum* | A3-1 (KT876717) | CRT |
|  | *Trichospoon inkin* | 7630 | CBS |
|  | *Trichospron loubieri* | 7065 | CBS |
|  | *Trichosporon mucoides* | 7625 | CBS |
|  |  |  |  |
| Zygomycota | *Lichtheimia corymbifera* | T14A (FJ713070) | CRT |
|  | *Mucor circinelloides* | E2A (FJ713065) | CRT |
|  | *Rhizomucar miehei* | MG4(2) (FJ713069) | CRT |
|  | *Rhizopus oryzae* | 112.09 | CBS |
|  | *Rhizopus stolonifer* var. *stolonifer* | 389.95 | CBS |
|  | *Saksenaea vasiformis* | 133.90 | CBS |
|  |  |  |  |
|  | ^a^ATCC; American Type Culture Collection, Teddington, UK.  CBS; Centraalbureau voor Schimmelcultures, Utrecht, The Netherlands.  CRT; C.R. Thornton, University of Exeter, UK.  IMI; International Mycological Institute, Wallingford, UK.  JL; J. Longcore, University of Maine, US.  MCF; M.C. Fisher, Imperial College London, UK.  NCPF; National Collection of Pathogenic Fungi, Salisbury, UK. | | |
