# Supplementary material for: Tracking the amphibian pathogens Batrachochytrium dendrobatidis and Batrachochytrium salamandrivorans using a highly specific monoclonal antibody and lateral‐flow technology
Source: Microb Biotechnol. 2016 Dec 19;10(2):381–94. doi: 10.1111/1751-7915.12464 (PMC5328824; doi:10.1111/1751-7915.12464)
Supplement: Supplementary file 2 — Table S2. Lateral‐flow assay test results for selected ELISA‐positive and ELISA‐negative fungi. [file MBT2-10-381-s002.docx]

| Table S2. Lateral-flow assay test results for selected ELISA-positive and ELISA-negative fungi. | | |
| --- | --- | --- |
| Organism | **Isolate no.** | **LFA result**^a^ |
|  |  |  |
| *Batrachochytrium dendrobatidis* | GPL 08MG02 | + |
| *Batrachochytrium dendrobatidis* | GPL JEL423 | + |
| *Batrachochytrium dendrobatidis* | GPL KBOOR317 | + |
| *Batrachochytrium dendrobatidis* | CAPE SA4c | + |
| *Batrachochytrium dendrobatidis* | SWISS 0739 | + |
| *Batrachochytrium dendrobatidis* | CAPE TF5a1 | + |
| *Batrachochytrium salamandrivorans* | 135744 | + |
| *Batrachochytrium salamandrivorans* | UK LFRC1 | + |
| *Homolaphlyctis polyrhiza* | JEL142 | + |
| *Allomyces macrogynus* | CBS221.89 | - |
| *Chytridium confervae* | CBS675.73 | - |
| *Phlyctochytrium reinboldtiae* | CBS669.73 | - |
| *Rhizophlyctis rosea* | CBS124.41 | - |
|  |  |  |
| *Saprolegnia diclina* | CBS113343 | - |
|  |  |  |
| *Candida albicans* | NGY152 | - |
| *Trichosporon asahii* var. *asahii* | CBS8973 | - |
| *Trichosporon asteroides* | CBS6183 | - |
| *Trichosporon inkin* | CBS7630 | - |
|  |  |  |
| ^a^ + positive (test line and internal control line visible); - negative (internal control line only) after 15 min. | | |
